# HIRD-Net: An Explainable CNN-Based Framework with Attention Mechanism for Diabetic Retinopathy Diagnosis Using CLAHE-D-DoG Enhanced Fundus Images

**DOI:** 10.3390/life15091411

**Published:** 2025-09-08

**Authors:** Muhammad Hassaan Ashraf, Muhammad Nabeel Mehmood, Musharif Ahmed, Dildar Hussain, Jawad Khan, Younhyun Jung, Mohammed Zakariah, Deema Mohammed AlSekait

**Affiliations:** 1 Faculty of Computing, Riphah International University, Islamabad 46000, Pakistan; hassaan.ashraf@riphah.edu.pk (M.H.A.); mnabeel.cs@gmail.com (M.N.M.); musharraf.ahmed@riphah.edu.pk (M.A.); 2 Department of Artificial Intelligence and Data Science, Sejong University, Seoul 05006, Republic of Korea; hussain.bangash@sejong.ac.kr; 3 School of Computing, Gachon University, Seongnam 13120, Republic of Korea; jkhanbk1@gachon.ac.kr (J.K.); younhyun.jung@gachon.ac.kr (Y.J.); 4 Department of Computer Science and Engineering, College of Applied Studies, King Saud University, P.O. Box 22459, Riyadh 11495, Saudi Arabia; mzakariah@ksu.edu.sa; 5 Department of Information Technology, College of Computer and Information Sciences, Princess Nourah bint Abdulrahman University, P.O. Box 84428, Riyadh 11671, Saudi Arabia; dmalsekait@pnu.edu.sa

**Keywords:** Computer-Aided Diagnosis, diabetic retinopathy diagnosis, fundus image enhancement, Difference of Gaussian, deep learning in medical imaging, Convolutional Neural Network, explainable AI, Grad-CAM, Hard-Swish

## Abstract

Diabetic Retinopathy (DR) is a leading cause of vision impairment globally, underscoring the need for accurate and early diagnosis to prevent disease progression. Although fundus imaging serves as a cornerstone of Computer-Aided Diagnosis (CAD) systems, several challenges persist, including lesion scale variability, blurry morphological patterns, inter-class imbalance, limited labeled datasets, and computational inefficiencies. To address these issues, this study proposes an end-to-end diagnostic framework that integrates an enhanced preprocessing pipeline with a novel deep learning architecture, Hierarchical-Inception-Residual-Dense Network (HIRD-Net). The preprocessing stage combines Contrast Limited Adaptive Histogram Equalization (CLAHE) with Dilated Difference of Gaussian (D-DoG) filtering to improve image contrast and highlight fine-grained retinal structures. HIRD-Net features a hierarchical feature fusion stem alongside multiscale, multilevel inception-residual-dense blocks for robust representation learning. The Squeeze-and-Excitation Channel Attention (SECA) is introduced before each Global Average Pooling (GAP) layer to refine the Feature Maps (FMs). It further incorporates four GAP layers for multi-scale semantic aggregation, employs the Hard-Swish activation to enhance gradient flow, and utilizes the Focal Loss function to mitigate class imbalance issues. Experimental results on the IDRiD-APTOS2019, DDR, and EyePACS datasets demonstrate that the proposed framework achieves 93.46%, 82.45% and 79.94% overall classification accuracy using only 4.8 million parameters, highlighting its strong generalization capability and computational efficiency. Furthermore, to ensure transparent predictions, an Explainable AI (XAI) approach known as Gradient-weighted Class Activation Mapping (Grad-CAM) is employed to visualize HIRD-Net’s decision-making process.

## 1. Introduction

**Figure 1:**
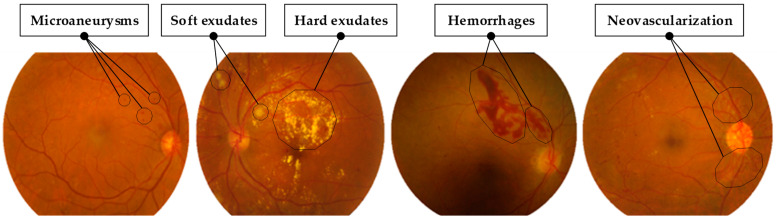
Typical Fundus Images (FIs) illustrating common DR lesions.

Diabetic Retinopathy (DR) is a critical medical condition and a leading cause of blindness, particularly among diabetic patients. It appears when high blood sugar levels damage the blood vessels in the retina [1]. Common symptoms of this retinal disease are illustrated in Figure 1, including microaneurysms appearing as small red dots, hemorrhages presenting as dark-red spots caused by ruptured blood vessels, and exudates categorized as either hard (yellow lipid deposits) or soft (cotton-wool patches indicating poor blood supply) [2]. Additionally, neovascularization indicates abnormal growth of new blood vessels, characteristic of proliferative DR, the disease’s advanced stage [3].

The World Health Organization predicts that by 2030, global diabetics will make up 4.4% of the population, and about half of them will experience DR complications [4]. This poses a significant burden on healthcare systems worldwide. Timely diagnosis of this retinal disease is crucial for effective treatment and prevention of further complications [5,6]. However, traditional methods of DR diagnosis, which often involve manual inspection by ophthalmologists, are not only time-consuming but also subject to human error [7,8]. This is especially problematic in remote or under-resourced settings, where access to specialized medical care is limited [2].

To address these challenges, CAD systems have been developed [4,7,9,10,11,12,13,14,15,16,17]. These systems typically follow one of two paradigms: traditional computer vision techniques or Deep Learning (DL) based methods [18]. Conventional approaches rely on handcrafted features [19], utilizing descriptors such as Local Binary Patterns (LBP) [20], Scale-Invariant Feature Transform (SIFT) [21], Bag of Words (BoW) [22], and Histogram of Oriented Gradients (HOG) [23] to extract visual cues from FIs. These features are then classified using machine learning algorithms like Support Vector Machines (SVM) [24], Naive Bayes (NB), or Random Forest (RF) [25]. While such pipelines can offer reasonable performance, their effectiveness is often constrained by the specificity of the engineered features [18,26]. Many of these descriptors are sensitive to variations in imaging conditions, patient demographics, and device types-leading to difficulties in generalizing across datasets. Moreover, conventional classifiers may struggle with high-dimensional or non-linear feature spaces, which can limit their robustness and diagnostic precision. The reliance on domain-specific feature engineering also makes these systems less adaptable to complex retinal pathologies, and subtle inter-class variations are commonly observed in this retinal disease [26].

The emergence of high-performance computing platforms such as Graphics Processing Units (GPUs) and Tensor Processing Units (TPUs) has significantly accelerated the adoption of DL models. These models are generally categorized into Vision Transformer (ViT) based and CNN-based approaches. In recent years, ViT frameworks have gained attention in biomedical image analysis. However, they often require extensive computational resources and large-scale labeled datasets, which limit their practical applicability in resource-constrained settings. This study focuses exclusively on CNN-based methods, with a strong emphasis on fundus image enhancement and hierarchical deep feature extraction. CNN-based classifiers like VGG-Net [27], InceptionNet [28], DenseNet [29], and ResNet [30] etc., have shown promising results in image classification tasks, including medical imaging [31]. In the domain of CNN-based medical image classification, VGG-Net [27] serves as a simple feed-forward neural network that employs fixed-sized filters (i.e., 3 × 3) for feature extraction. However, the features extracted in each layer are exclusively used in the subsequent layer only, limiting the network’s ability to propagate information efficiently across consecutive layers [32]. In contrast, InceptionNet [28] employs multiple-sized filters, allowing for feature extraction at different scales (i.e., 3 × 3, 5 × 5), thereby offering greater flexibility in capturing complex patterns [33]. DenseNet [29] and ResNet [30] take this a step further by utilizing Multi-Level Features (MLF) and implementing advanced mechanisms for more efficient information propagation within the network. Specifically, DenseNet employs dense connections between layers to maximize information flow [34], while ResNet uses residual connections to facilitate the training of deeper networks and mitigate the vanishing gradient problem [25].

However, these general CNN-based classifiers face multiple challenges in real-time DR diagnosis applications. These challenges include early-stage diagnosis, disease class variation, limited samples in benchmark datasets, high computational costs, as well as lesion scale variation, class variations, and gradient vanishing [2,6]. Each of these factors adds a layer of complexity to the diagnostic process, making it difficult to develop a universally effective and efficient system.

To overcome these limitations, this research introduces a novel CNN-based framework designed for automated DR diagnosis. The proposed approach employs a robust preprocessing pipeline to enhance the quality and diagnostic features of retinal images. This preprocessing strategy integrates CLAHE, effectively improving image contrast, with Difference of Gaussian (DoG) filtering to highlight detailed retinal structures and mitigate blurry morphological patterns. The proposed framework’s backbone architecture, HIRD-Net, incorporates a hierarchical feature extraction stem with multiscale and multilevel inception-residual-dense blocks. To further refine learned representations, the features extracted from each module are passed through an attention block, which emphasizes the most salient retinal patterns. It is further enhanced by the Hard-Swish activation function to improve gradient flow stability. The Softmax activation function serves as a classifier within the CNN, and the focal loss function is utilized to mitigate the class imbalance issue. Additionally, four GAP layers enable deeper semantic aggregation across multiple scales, significantly improving classification accuracy and reducing overfitting risks. To ensure interpretable decision-making, the Grad-CAM approach is integrated to visualize the regions most influential in the predictions. Moreover, data augmentation techniques validate the robustness and adaptability of HIRD-Net across datasets of varying sizes, ensuring both scalability and generalization in practical diagnostic scenarios.

This research offers several significant contributions to overcome the limitations of current CAD systems in DR diagnosis:

- Integration of CLAHE with D-DoG filtering for enhanced image preprocessing, effectively addressing blurred morphological patterns and improving the visibility of fine-grained features in FIs.
- Proposal of a novel CNN-based architecture, HIRD-Net, specifically designed for DR diagnosis. The model incorporates a hierarchical feature extraction stem along with multiscale and multilevel blocks, enabling the capture of subtle and diverse pathological features. The architecture utilizes four GAP layers to enhance semantic feature aggregation and mitigate overfitting. The Hard-Swish activation function is employed to stabilize gradient propagation, while Softmax activation, combined with focal loss and extensive data augmentation, is used to effectively address class imbalance.
- Incorporate Grad-CAM to provide visual interpretability of HIRD-Net predictions, enabling transparent decision-making and highlighting pathological regions that influence classification.
- Comprehensive empirical evaluation by comparing the proposed framework with existing state-of-the-art methods, demonstrating superior performance across key metrics, including precision, recall, F1-score, and accuracy.

The remainder of this paper is structured to provide a comprehensive overview of the proposed framework and its application to DR diagnosis. Section 2 presents a critical review of related work, outlining current methodologies and identifying key challenges in the field. Section 3 details the preprocessing strategy, the architecture of HIRD-Net, and the function of each component within the framework. Section 4 discusses the experimental set-up and presents an ablation study to evaluate the contribution of individual modules. Section 5 provides a comparative analysis with existing models and discusses the findings in the context of model performance and clinical relevance. Finally, Section 6 concludes the paper and outlines potential directions for future research.

## 2. Current State-of-the-Art

The application of CNN in the diagnosis of DR has been a subject of extensive research. Several studies have employed different CNN architectures to address the challenges associated with this retinal disease diagnosis.

Islam et al. [35] proposed a Supervised Contrastive Learning (SCL) framework for diagnosing DR using FIs. They used the Xception-Net CNN architecture for feature extraction. They also applied a CLAHE-based preprocessing technique to improve the quality of the retinal scans and increased the data samples by applying rotation of 90° and 270°, as well as vertical and horizontal flipping. For testing the performance of their framework, they used two public benchmarks: APTOS2019 and Messidor-2. Nahiduzzaman et al. [3] proposed a parallel CNN framework. They improved the input retinal images by applying the CLAHE image enhancement technique. This technique makes the DR lesions clearer. They used four parallel CNN architectures with different kernel sizes (3 × 3, 5 × 5, 7 × 7, 9 × 9) for feature extraction. These different scale features were then combined for final feature extraction. To evaluate their framework, they used the Kaggle DR 2015 and APTOS2019 datasets.

Khan et al. [36] leveraged the VGG16 architecture as a baseline to develop a scale-invariant diagnosis framework, termed VGG-NiN. One of the key innovations in their approach was the introduction of a Spatial Pyramid Pooling (SPP) block before the Fully Connected (FC) layer. This SPP block was designed to extract Multi-Scale Features (MSF), thereby enhancing the model’s ability to handle variations in lesion scales. Similarly, da Rocha et al. [10] utilized the VGG16 architecture but incorporated transfer learning techniques for this chronic disease diagnosis. They initially trained the VGG16 model using weights from the ImageNet dataset, adapting it subsequently for DR diagnosis. This approach leveraged the pre-trained model’s ability to recognize complex features, thus potentially improving the diagnosis accuracy. Yaqoob et al. [25] proposed a hybrid approach that utilized the deep learning features of a fine-tuned ResNet-50 model. These features were then fed into a RF classifier for the final classification. This method notably bypasses the conventional use of FC layers, offering a different idea for features classification. Deng et al. [37] took a slightly different approach by employing both VGG16 and ResNet50 architectures for retinal FI analysis. One of their significant contributions was the reduction of the FC layers in the VGG16 architecture. This modification aimed to decrease the overall number of parameters in the CNN architecture, thereby making the model more computationally efficient.

Lin et al. [38] focused on the ResNet50 architecture for DR diagnosis. Their work introduced two key innovations: a Standard Operating Procedure (SOP) for preprocessing FIs and a revised structure of ResNet-50. The latter included an adaptive learning rate, layer regularization, and structural modifications to ResNet-50. Kobat S.G. et al. [34] leveraged the DenseNet201 architecture, incorporating transfer learning for DR image classification. Their approach introduced a patch division method, segmenting images into horizontal and vertical patches to improve classification accuracy. While the densely connected blocks in DenseNet201 facilitated MLF extraction, they also increased the model’s complexity, posing challenges such as overfitting and higher computational demands. Gangwar and Ravi [14] introduced a hybrid model that combined the Inception-ResNet-v2 architecture with a custom block of CNN layers. The Inception component was crucial for MSF extraction, while the ResNet component facilitated the extraction of MLF. This hybrid model demonstrated superior performance on the Messidor-1 and APTOS 2019 datasets compared to other state-of-the-art models.

Tariq et al. [31] explores the application of deep transfer learning to the diagnosis of DR by leveraging five different CNN architectures: AlexNet, GoogleNet, Inception V4, Inception ResNet V2, and ResNeXt-50. A dataset of DR images was compiled and labeled with corresponding treatment approaches, thereby facilitating the automation of diagnosis and aiding in the management of subsequent therapies. The work employs a private dataset to train these deep CNNs for grading the severity of DR in retinal images. Among the tested pre-trained models, Se-ResNeXt-50 demonstrates the highest classification accuracy, underscoring the potential of this model in enhancing DR diagnosis through DL techniques. Saranya et al. [39] developed a CNN model structured with three integral blocks, including convolution layers, batch normalization, and the ReLU activation function. They integrated a dropout layer to improve the model’s efficacy. Their methodology also included the segmentation of the optic disk before analysis to avoid misclassification, and they employed various preprocessing techniques like Canny Edge detection.

The above literature on the use of CNN for DR diagnosis presents a variety of architectures and methodologies, each contributing uniquely to the field; however, several critical gaps remain. Notably, current studies often overlook the integration of effective preprocessing methods that enhance image quality and diagnostic features, essential for accurate diagnosis. Moreover, there are noticeable gaps in handling MSF and MLF simultaneously, computational efficiency, and robust performance across varied dataset sizes. While some models focus on MSF extraction, they often do not adequately integrate MLF, potentially leading to suboptimal performance [2]. Furthermore, there are challenges related to the computational efficiency of complex models, as well as inconsistent performance when transitioning between large and small datasets. The proposed framework aims to fill these gaps by proposing an architecture that seamlessly combines MSF and MLF, and maintains computational efficiency, and ensures consistent performance across different dataset sizes, thus addressing the limitations found in the current literature.

## 3. Materials and Methods

**Figure 2:**
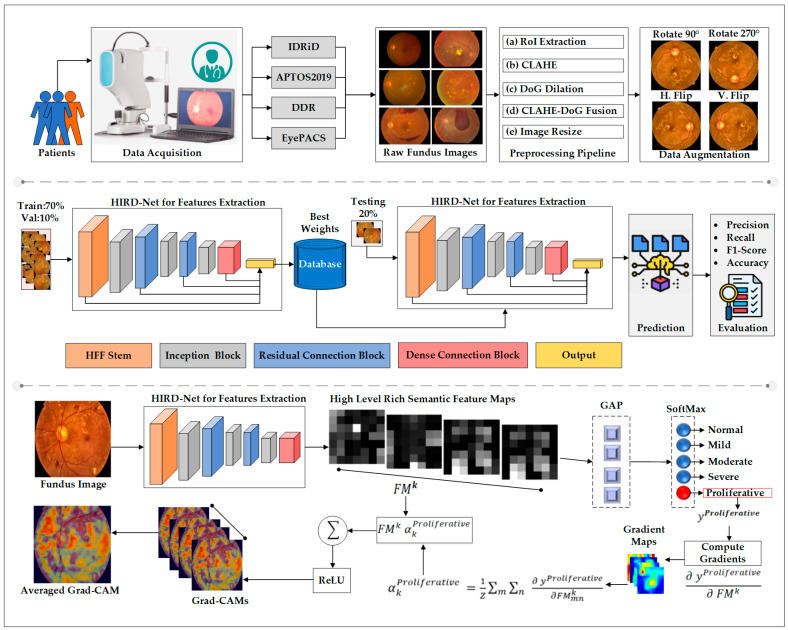
Overview of the proposed XAI-based DR diagnostic pipeline.

The proposed framework introduces a comprehensive solution for DR diagnosis using FIs. This section describes the key components of the framework, including the experimental datasets, preprocessing and enhancement pipeline, and data augmentation techniques implemented to improve input diversity. The architecture of the HIRD-Net model is outlined in detail, highlighting its hierarchical and multiscale design. Additionally, the selection and role of activation and loss functions are discussed. An overview of the complete diagnostic pipeline is illustrated in Figure 2.

### 3.1. Experimental Datasets

This research utilized four publicly available benchmark datasets, IDRiD [40], APTOS2019 [41], DDR [42], and EyePACS [43] to validate the performance of the proposed framework. The IDRiD dataset comprises 516 high-resolution color FIs, APTOS2019 includes 3663 images, DDR consists of 12,522 retinal samples, and EyePACS contains 35,126 images categorized into five classes: (i) Normal, (ii) Mild, (iii) Moderate, (iv) Severe, and (v) Proliferative DR. To enhance evaluation robustness, this study also combined the IDRiD and APTOS2019 datasets, resulting in a total of 4178 images.

### 3.2. Data Preprocessing and Enhancement

**Figure 3:**
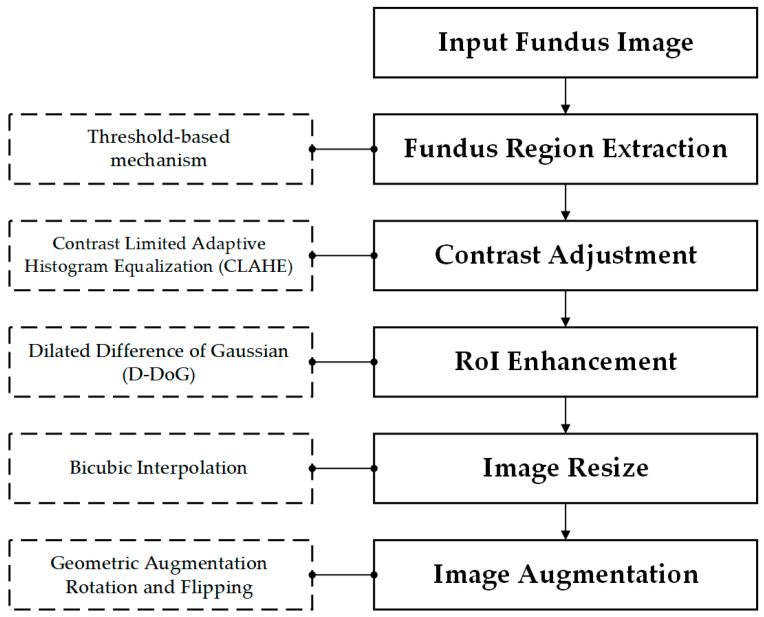
Data preprocessing and preparation pipeline.

To optimize the visual quality and diagnostic richness of retinal images, this research developed a multi-stage preprocessing and enhancement pipeline, as illustrated in Figure 3.

**Figure 4:**
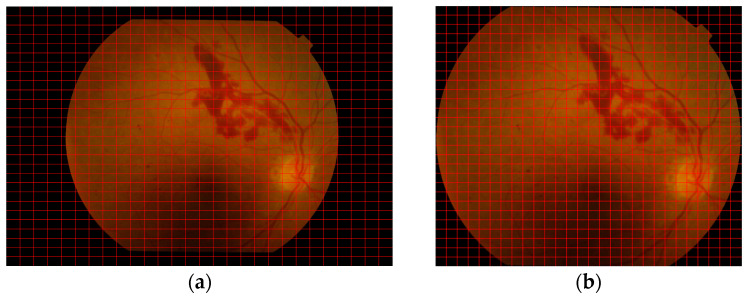
(**a**) Original Image (2896 × 1944), (**b**) Cropped Image (2057 × 1749).

Initially, we focused on isolating the fundus region by removing the surrounding blank boundary areas. We achieved this using a simple yet effective threshold-based segmentation approach [2], which efficiently excluded non-fundus regions. We first converted the input image to grayscale to reduce computational complexity and simplify the subsequent analysis. We then applied thresholding by treating pixel values less than 10 as background, effectively suppressing low-intensity artifacts and enhancing the visibility of the retinal region. To localize the area of interest, contour analysis was performed, with the largest detected contour assumed to represent the retinal boundary-based on the assumption that the retina occupies the most prominent region in the image. We then computed a bounding box around this contour and cropped the original RGB image accordingly. The resulting cropped image, focused solely on the fundus area, was saved for downstream analysis. Figure 4 illustrates an example of the original image and the final cropped retinal image.

**Figure 5:**
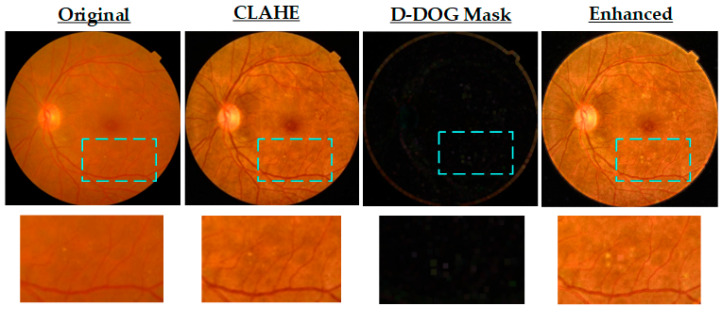
Visualization of FI enhancement process using CLAHE and D-DoG filtering.

We subsequently processed the cropped images using contrast adjustment and enhancement. This enhancement utilizes CLAHE with a clip limit of 4.0 and a tile grid size of 8 × 8 to improve local contrast. These parameters were empirically selected to strike a balance between enhancing subtle retinal features and minimizing noise amplification. A higher clip limit intensifies local contrast variations, aiding in the visualization of poorly illuminated or subtle pathological regions, while a larger tile grid captures broader contextual information, promoting uniform contrast across the fundus. To further enhance lesion-level structures and suppress low-frequency background textures, we applied a DoG filter to the CLAHE-enhanced image. This study applied a 9 × 9 Gaussian kernel with a fixed σ = 2.0 to smooth the image, aligning with the approximate spatial scale of small retinal lesions such as microaneurysms and hemorrhages. We computed the DoG mask by subtracting the blurred image from the CLAHE image, effectively isolating high-frequency pathological features. To further amplify these features, we performed morphological dilation on the DoG mask using a 2 × 2 structuring element with a single iteration. This operation gently expands feature boundaries without causing over-segmentation. We then replicated the resulting D-DoG mask across three channels and additively fused with the CLAHE-enhanced image. The final composite image thus integrates global contrast enhancement with localized detail preservation. Figure 5 illustrates the complete enhancement pipeline, showing the original mild DR image, the CLAHE-enhanced image, the D-DoG mask, and the final CLAHE-D-DoG enhanced image. Colored frames highlight ROIs before and after preprocessing, illustrating how CLAHE and D-DoG integration enhances the visibility of fine retinal structures compared to the original image.

**Figure 6:**
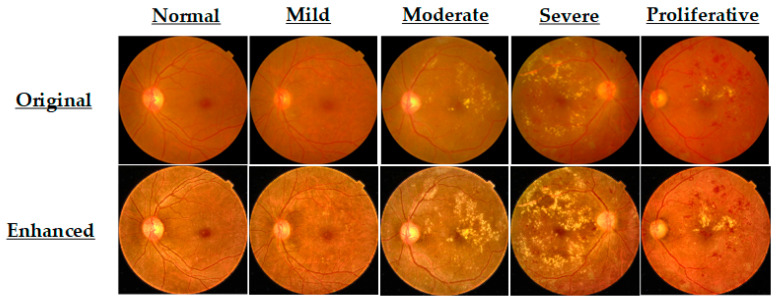
Visual comparison of original and CLAHE-D-DoG enhanced retinal images.

This fusion enhances both vascular networks and lesion-level cues essential for accurate DR diagnosis. Representative enhanced FI samples from each DR severity class are presented in Figure 6.

Following contrast enhancement and lesion refinement, we standardized the input image dimensions to 224 × 224 pixels using bicubic interpolation. This resizing step ensures compatibility with the input requirements of the proposed HIRD-Net architecture. We utilized bicubic interpolation over simpler methods such as nearest neighbor or bilinear interpolation due to its ability to produce smoother and more visually coherent results, particularly around fine edges and lesion boundaries [2]. Unlike nearest-neighbor (which can introduce pixelation) or bilinear (which may blur high-frequency features), bicubic interpolation leverages a 4 × 4 neighborhood of surrounding pixels, preserving the anatomical and pathological structures emphasized during preprocessing.

**Figure 7:**
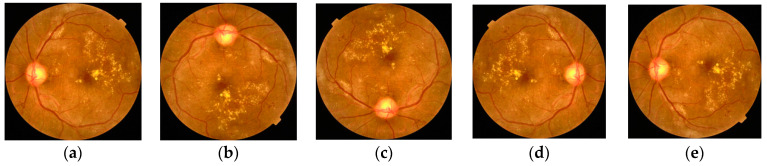
(**a**) Enhanced image, (**b**) Rotate at 90°, (**c**) Rotate 270, (**d**) Horizontal Flip, (**e**) Vertical Flip.

Following enhancement and resizing, the images of the combined dataset (i.e., IDRiD and APTOS2019) were subjected to several geometric transformations, including 90° rotation, 270° rotation, horizontal flipping, and vertical flipping, as illustrated in Figure 7. For the DDR and EyePACS datasets, which exhibit significant class imbalance, geometric minority oversampling was applied to the Mild, Severe, and Proliferative DR classes to mitigate the imbalance problem. The same set of geometric transformations was applied to augment these minority classes. For the binary classification evaluation, the combined datasets are restructured such that all Class 0 samples (*n* = 9865) are designated as Normal, while all Class 1–4 samples (*n* = 11,025) are aggregated as Abnormal.

After preprocessing and augmentation, we split the dataset into 70% for training, 10% for validation, and 20% for testing. A detailed summary of the final preprocessed dataset used in this study is presented in Table 1.

### 3.3. HIRD-Net for Features Extraction and Classification

**Figure 8:**
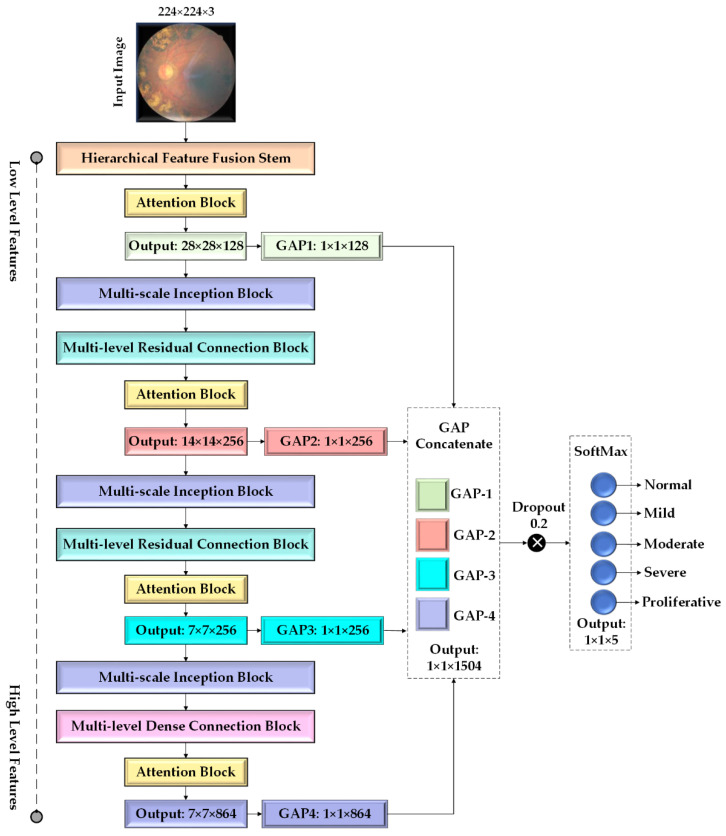
Proposed HIRD-Net architecture for DR diagnosis.

This research proposes a novel CNN architecture, HIRD-Net, which integrates the advantages of various CNN architectures to develop a robust model that balances processing speed and classification performance. Figure 8 illustrates the HIRD-Net architecture designed for DR diagnosis.

**Figure 9:**
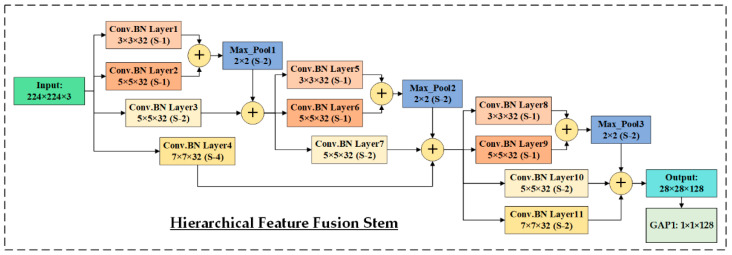
Proposed Hierarchical Features Fusion Stem employed in HIRD-Net.

The HIRD-Net is designed to process color FIs with input dimensions of 224 × 224 × 3 (width, height, color channels). The input is first passed through a specialized Hierarchical Feature Fusion (HFF) stem shown in Figure 9, which differs significantly from traditional Simple Feed Forward (SFF) stems that typically use shallow, feed-forward layers. The HFF stem is architected to extract MSF at varying receptive fields and fuse them hierarchically across different depth levels, promoting rich and discriminative representations.

Initially, the input is processed through four parallel convolutional branches, each comprising Convolution-Batch Normalization (Conv.BN) layers with 32 filters. Specifically, Conv.BN Layer1 and Layer2 use 3 × 3 and 5 × 5 filters (stride 1), and their outputs are concatenated. The concatenated features are down-sampled using Max_Pool1 (2 × 2, stride 2), yielding 112 × 112 × 64 FMs. Simultaneously, the input image is passed through Conv.BN Layer3 (5 × 5, stride 2) and Layer4 (7 × 7, stride 4). The output of Layer3 is concatenated with the output of Max_Pool1 to form a 112 × 112 × 96 FMs, which is then processed by Conv.BN Layers 5–7, repeating the fusion strategy with updated filter sizes and strides. This results in 56 × 56 × 96 FMs, which are further concatenated with the output of Layer4, forming a 56 × 56 × 128 tensor. This intermediate representation is passed through another set of convolutional layers: Layer8 (3 × 3, stride 1), Layer9 (5 × 5, stride 1), Layer10 (5 × 5, stride 2), and Layer11 (7 × 7, stride 2), producing rich semantic features of dimensions 28 × 28 × 128.

**Figure 10:**
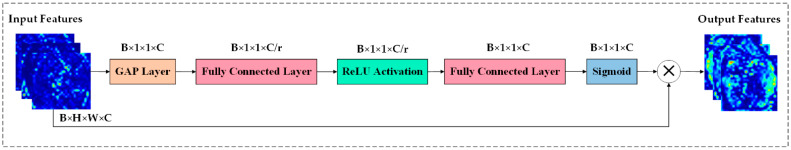
Squeeze-and-Excitation Channel Attention (SECA) Block employed in HIRD-Net.

Following hierarchical feature aggregation, the semantic features are refined using an attention block based on the Squeeze-and-Excitation Channel Attention (SECA) mechanism [44], as illustrated in Figure 10. This module adaptively re-weights channel responses to enhance lesion-relevant features. Given an input FM $x\in R^{B\times H\times W\times C}$ where *B* denotes the batch size (e.g., 32), *H* and *W* are the spatial dimensions, and *C* is the number of channels, a GAP operation (orange block) compresses the spatial dimensions for each image in the batch to produce a channel descriptor $z\in R^{B\times 1\times 1\times C}$. This descriptor is passed through a FC layer (i.e., first pink block) followed by a ReLU activation as shown in the green block, reducing the dimensionality to *B* × 1 × 1 × (*C*/*r*) (with *r* = 8). After activation, these features are passed through a second pink block FC layer that restores the original dimensionality and applies a sigmoid activation as shown in blue block, yielding channel attention weights. The recalibrated FM is then obtained via element-wise multiplication between the channel attention weights and the original FM. This channel-wise recalibration biases the network towards channels encoding diagnostic cues such as microaneurysms, hemorrhages, and hard exudates.

**Figure 11:**
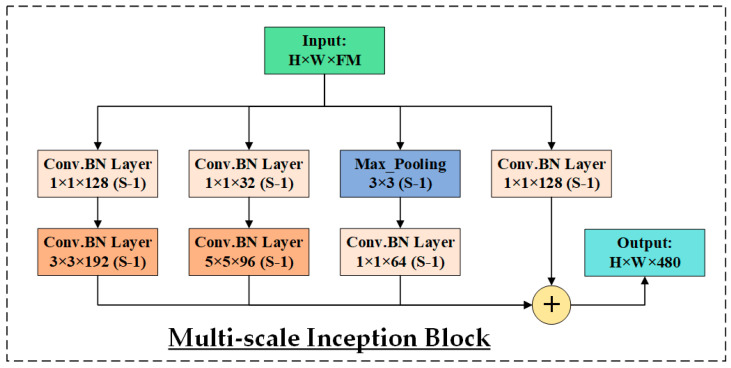
Multi-scale Inception Block employed in HIRD-Net.

The refined features (28 × 28 × 128) from the attention block are fed into the first GAP layer, reducing the FMs to 1 × 1 × 128. This operation captures the global context required for the final classification. In addition, these features serve as the input to the subsequent multi-scale Inception-like block, as illustrated in Figure 11, to effectively capture retinal features that manifest at varying spatial scales. DR symptoms may appear as subtle, fine-grained microaneurysms or as large, diffuse hemorrhages and exudates. Therefore, extracting multi-scale information is crucial for accurately classifying different stages of the disease. The inception module addresses this by processing the input through four parallel convolutional pathways, each tailored to extract features at different granularities.

The first branch applies a 1 × 1 convolution with 128 filters to capture fine local patterns and reduce dimensionality. The second branch also begins with a 1 × 1 convolution (128 filters), followed by a 3 × 3 convolution (192 filters). This pathway is designed to capture mid-level spatial structures and edge transitions. The third branch starts with a 1 × 1 convolution (32 filters) and is followed by a 5 × 5 convolution (96 filters), which targets larger, more global retinal patterns relevant to advanced DR stages. The fourth branch performs 3 × 3 max pooling, which helps retain dominant spatial features while reducing spatial resolution. This is followed by a 1 × 1 convolution with 64 filters to recover channel depth and integrate local activations. All four branches are concatenated along the channel dimension, producing a comprehensive MSF map of size H × W × 480. This diverse representation allows HIRD-Net to learn robust, discriminative features for DR classification across varying severity levels.

**Figure 12:**
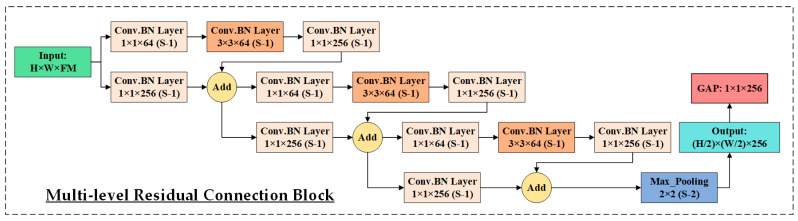
Multi-level Residual Connection Block (RCB) employed in HIRD-Net.

The output of the Inception Block (28 × 28 × 480) is then passed through the multi-level Residual Connection Block (RCB), as shown in Figure 12. This block is intended to extract deep, MLF while addressing the vanishing gradient problem and promoting efficient information flow through the network.

**Figure 13:**
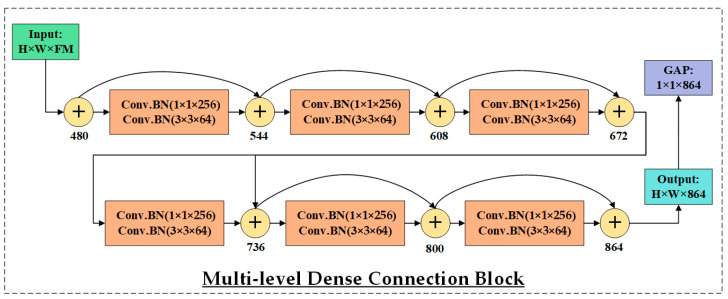
Multi-level Dense Connection Block (DCB) employed in HIRD-Net.

The RCB begins with a 1 × 1 Conv.BN layer to project the input into a lower-dimensional space. This is followed by a 3 × 3 Conv.BN layer to extract 64 FMs, and a final 1 × 1 Conv.BN layer to restore or adjust the channel depth while preserving the spatial dimensions. This sequence forms a residual unit, which is repeated three times to progressively learn deeper and more abstract representations. The RCB incorporates multiple identity sub-blocks, each replicating this structure but maintaining both spatial resolution and FMs depth. To facilitate residual learning, shortcut connections are included in each sub-block, allowing the original input to bypass the intermediate layers. In cases where the input and output dimensions do not match, a 1 × 1 convolution is applied to align them before addition. This residual design enables stable gradient flow during backpropagation, thereby allowing the construction and effective training of deeper networks. The output of the RCB is a 28 × 28 × 256 FMs rich in semantic information. It is then down-sampled using a 2 × 2 max pooling layer (stride = 2), resulting in a 14 × 14 × 256 FMs. This representation is subsequently passed through an attention block and routed to both the second GAP layer and a second multi-scale Inception Block. The latter produces 14 × 14 × 480 features, which are then fed into a second RCB for further deep feature extraction. The second RCB produces a 7 × 7 × 256 feature representation. This output is refined through a third attention block and is concurrently routed to the third GAP layer for semantic aggregation, as well as to a third Multi-Scale Inception Block for further multi-resolution feature extraction. The output of this module is a 7 × 7 × 480 FMs that encapsulates a rich and diverse set of spatial representations. This representation is then fed into the Multi-Level Dense Connection Block (DCB) as illustrated in Figure 13, which serves as a key component in HIRD-Net’s deep feature learning strategy.

The DCB, inspired by DenseNet architecture, is designed to address core challenges such as limited feature reuse and vanishing gradients in deep networks. By adopting dense connectivity, the DCB enables each layer to access the outputs of all preceding layers, promoting efficient feature propagation and learning continuity. It consists of six densely connected convolutional units, each composed of batch normalization, a 1 × 1 convolution with 256 filters for dimensionality adaptation, followed by another batch normalization and a 3 × 3 convolution with 64 filters for spatial feature extraction. The cumulative concatenation of outputs from all layers results in a final tensor of size 7 × 7 × 864. This deep, semantically rich representation is then passed through a final attention block and a fourth GAP layer, producing a compact 1 × 1 × 864 descriptor.

**Figure 14:**
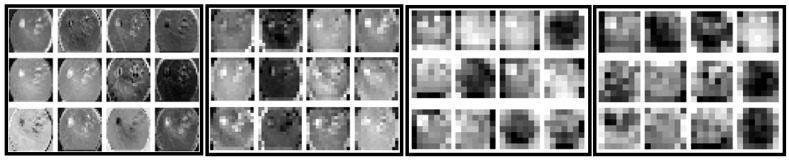
Representative FMs samples fed into the GAP-1 to GAP-4 layers (from left to right).

To provide insights into the nature of the features contributing to the final classification, Figure 14 illustrates representative samples of FMs passed through each GAP layer. These visualizations help clarify the scale and abstraction level of the inputs feeding into each GAP operation. As shown, the shallow layers (GAP-1 and GAP-2) capture low-level features and edge-related information, while deeper layers (GAP-3 and GAP-4) progressively capture high-level rich semantic patterns related to pathological cues.

The outputs from all four GAP layers, each capturing discriminative features at different spatial depths, are then concatenated in the final GAP concatenation unit. This design ensures that the classification layer receives comprehensive, hierarchical, MLF, and MSF representation, leveraging both low-level and high-level cues for robust DR diagnosis.

To improve generalization and reduce overfitting, a dropout regularization strategy is employed before the final classification. A dropout rate of 0.2 is applied, meaning that 20% of the neurons are randomly disabled during each training iteration. This prevents the model from relying too heavily on specific features, encouraging it to learn more distributed and generalized representations.

#### 3.3.1. Activation Function

The activation function is an essential component of CNNs, introducing non-linearity to facilitate the learning of complex representations. While widely used functions such as Tanh and ReLU have been effective, they exhibit notable limitations. Tanh suffers from gradient vanishing, especially in deeper networks, and ReLU is prone to producing dead neurons due to zero gradients for negative inputs, leading to FM sparsity and training inefficiency [2].

To address these issues, the Swish activation function was previously employed in DR diagnosis [2] which is formulated in Equation (1).

(1) $$f\left(x\right)=x\cdot Sigmoid\left(x\right)=\frac{x}{1+e^{x}}\;$$

Swish activation function enables smoother gradient flow and has been shown to improve convergence and model accuracy. However, due to its reliance on the computationally expensive sigmoid function, Hard-Swish, a more efficient approximation of Swish, has been adopted in our proposed HIRD-Net.

The Hard-Swish activation is expressed in Equation (2).

(2) $$HardSwish\left(x\right)=x\cdot \frac{ReLU6\left(x+3\right)}{6}$$

where Equation (3) describes ReLU6.

(3) $$ReLU6\left(x\right)=\min\left(max\left(0,\;x\right),6\right)$$

This function is both piecewise linear and computationally lightweight, making it particularly well-suited for deep networks. Compared to ReLU, Tanh and Swish, Hard-Swish retains the smoothness and gradient-friendly properties of Swish while achieving greater computational efficiency. In HIRD-Net, Hard-Swish is employed across all major building blocks to enhance learning stability, improve gradient propagation, and ensure the activation of a broader range of neurons.

#### 3.3.2. Loss Function

In the context of DR diagnosis, the datasets often exhibit significant class imbalance, which biases standard loss functions like cross-entropy toward the majority classes, resulting in poor sensitivity for minority class detection. To address this challenge, HIRD-Net employs the Focal Loss function, which is specifically designed to handle severe class imbalance by down-weighting easy examples and focusing the training on hard, misclassified samples [32]. For a multi-class classification problem, the focal loss is explained in Equation (4).

(4) $$L\left(Y_{act},f\left(x\right)\right)=-\frac{1}{N}\sum_{i=1}^{N}\;\sum_{j=1}^{C}\;\beta_{j}{\left(1-f_{j}(x_{i})\right)}^{\alpha }\;y_{ij}log(f_{j}\left(x_{i};\theta \right))$$

where $Y_{act}$ denotes the actual class labels and f(*x*) represents the predicted probabilities. *N* is the total number of training samples, and *C* denotes the number of classes (i.e., *C* = 5). While $y_{ij}$ is the ground truth indicator, where $y_{ij}$=1 if sample *i* belongs to class j, and 0 otherwise. $f_{j}$ is the predicted probability for class j, $\beta_{j}$ is a weighting factor used to balance class contributions, and *α* ≥ 0 is the focusing parameter that down-weights the loss assigned to well-classified examples, thereby placing greater emphasis on hard or misclassified samples.

### 3.4. Interpretability Analysis Using Grad-CAM

The proposed framework employs Gradient-weighted Class Activation Mapping (Grad-CAM) [45] as an explainable AI (XAI) approach to generate class-specific localization maps, highlighting regions of the input FI most influential in the prediction. In HIRD-Net, Grad-CAM is applied to the final convolutional block preceding the fourth GAP layer.

Given an input FI and a target class c, the gradient of the class score $y^{c}$ with respect to the feature maps $FM^{k}$ of the selected convolutional layer is computed. The weight $\alpha_{k}^{c}$ for each feature map channel k is computed as shown in Equation (5).

(5) $$\alpha_{k}^{c}=\frac{1}{H\times W}\sum_{i=1}^{H}\;\sum_{j=1}^{W}\;\frac{\partial \;y^{C}}{\partial FM_{i,j}^{k}}$$

The Grad-CAM is derived according to Equation (6).

(6) $$L_{Grad-CAM}^{c}=ReLU\sum_{i=1}^{H}\alpha_{k}^{c}\;FM^{k}\;\;$$

where H and w denote the dimension of FMs and the ReLU ensure that only features exerting a positive influence on the target class are preserved. The resulting heatmap visually emphasizes pathological regions such as microaneurysms, hemorrhages, and exudates that contribute significantly to the model’s decision.

### 3.5. Model Training and Testing

The training and evaluation of HIRD-Net were conducted using an NVIDIA Tesla T4 GPU. The Tesla T4 features 40 streaming multiprocessors, a 6 MB shared L2 cache, and 16 GB of GDDR6 memory. With a clock speed of 1.59 GHz, 64 FP32 arithmetic units per core, and a maximum memory bandwidth of 300 GB/s, the GPU delivers up to 8.1 TFLOPS of performance for FP32 computations and 65 TFLOPS for FP16 precision.

In this research, during HIRD-Net training, a batch size of 32 was employed to maintain a balance between memory efficiency and gradient stability. The AdamW optimizer [46] was employed due to its adaptive learning rate mechanism and decoupled weight decay regularization, which together promote faster convergence, improved generalization, and more stable training dynamics. All models were trained for 90 epochs, ensuring adequate learning time for capturing complex patterns in the FIs. During training, we implemented a model checkpointing strategy, whereby the weights yielding the best performance on the validation set were automatically saved. This approach ensured that the most optimal version of each model was preserved for final evaluation. In the testing phase, we used the saved best-performing weights to assess each model’s generalization ability and classification accuracy. This ensured that the reported results reflect the peak performance of each CNN, including HIRD-Net, in classifying FIs across multiple DR severity levels.

### 3.6. Performance Evaluation Metrics

We assessed the effectiveness of the proposed framework, along with other classifiers [28,29,30], using various weighted metrics such as recall, precision, F1-score, and overall accuracy. The adoption of weighted precision, recall, and F1-score is justified by their relevance to real-world scenarios. Misclassifications in larger classes can have more substantial practical implications than errors in smaller classes. Therefore, weighted metrics provide a nuanced evaluation, emphasizing the importance of each class’s size. Additionally, these metrics offer a balanced assessment in the presence of dataset imbalances, where certain conditions might be underrepresented. Traditional macro metrics might not fully reflect the model’s performance in such cases. In contrast, weighted metrics ensure a more comprehensive evaluation by proportionally accounting for the prevalence of each class [2].

These standard classification metrics are derived from the confusion matrix (CM), as shown in Equations (7)–(13). The matrix entry at position CM[i][j] indicates the number of instances belonging to class i that have been predicted as class j.

- True Positives (TP) for class i are denoted by CM[i][i].
- False Positives (FP) for class i are calculated as the sum of instances incorrectly predicted as class i, i.e., $\sum_{j=1,\;j\neq i}^{n}CM[j][i]$.
- False Negatives (FN) for class i correspond to samples that actually belong to class i but are misclassified as other classes, i.e., $\sum_{j=1,\;j\neq i}^{n}CM[i][j]$.

Based on these definitions, the following class-wise metrics are computed:

(7) $${Precision}_{i}=\frac{{TP}_{i}}{{TP}_{i}+{FP}_{i}}\;=\;\frac{CM[i][i]\;}{CM[i][i]+\sum_{j\neq i}CM[j][i]},$$

(8) $${Recall}_{i}=\frac{{TP}_{i}}{{TP}_{i}+{FN}_{i}}=\frac{CM[i][i]\;}{CM[i][i]+\sum_{j\neq i}CM[i][j]},$$

(9) $$Weighted\;Precision=\;\sum_{i=1}^{n}\left(\frac{CM[i][i]\;}{CM[i][i]+\sum_{j=1,\;\;j\neq i}^{n}\sum_{j=1}^{n}CM[j][i]}\right)\times \frac{\sum_{j=1}^{n}CM[i][j]\;}{\sum_{i=1}^{n}\sum_{j=1}^{n}CM[i][j]},$$

(10) $$Weighted\;Recall=\;\sum_{i=1}^{n}\left(\frac{CM[i][i]\;}{CM[i][i]+\sum_{j=1,\;\;j\neq i}^{n}\sum_{j=1}^{n}CM[i][j]}\right)\times \frac{\sum_{j=1}^{n}CM[i][j]\;}{\sum_{i=1}^{n}\sum_{j=1}^{n}CM[i][j]},$$

(11) $${F1}_{i}=\;\frac{2\times {Precision}_{i}\times {Recall}_{i}}{{Precision}_{i}+{Recall}_{i}},$$

(12) $$Weighted\;F1\;Score=\sum_{i=1}^{n}{F1}_{i}\times \frac{\sum_{j=1}^{n}CM[i][j]\;}{\sum_{i=1}^{n}\sum_{j=1}^{n}CM[i][j]},$$

(13) $$Accuracy=\frac{\sum_{i=1}^{n}CM[i][i]\;}{\sum_{i=1}^{n}\sum_{j=1}^{n}CM[i][j]},$$

where n represent the total classes.

## 4. Results

This section explores the experimental results of the proposed framework across multiple publicly available datasets.

### 4.1. Ablation Study on IDRiD and APTOS2019

To evaluate the contribution of each component within the proposed framework, we conducted a series of ablation experiments. These experiments were designed to assess the impact of architectural modifications, feature extraction strategies, and preprocessing techniques. While multiple configurations were tested, only the most significant and representative results are discussed here. A summary of the ablation results is presented in Table 2.

We performed two types of experiments using publicly available datasets to comprehensively evaluate the proposed framework. We formulated the first task, DR screening, as a binary classification problem to distinguish healthy from unhealthy FIs. The second type, DR grading, involved a more complex multi-class classification task, where the goal was not only to detect the presence of DR but also to accurately identify its severity stage. These two experimental setups allowed for a robust assessment of the model’s capability in both disease detection and detailed severity classification.

In the initial stage of ablation, we explored the influence of incorporating MSF and MLF. Well-known CNN architectures such as ResNet-50, DenseNet-201, and InceptionNet were used as baselines. These models adopt a simple feed-forward (SFF) stem, where features are extracted sequentially without hierarchical fusion. Without preprocessing or augmentation, ResNet-50 and DenseNet-201 (which both support MLF but not MSF) achieved screening accuracies of 93.13% and 95.88%, and grading accuracies of 61.79% and 68.56%, respectively. InceptionNet, which emphasizes MSF but lacks MLF, outperformed both with 96.72% (screening) and 72.01% (grading). To test the synergy of MSF and MLF, we introduced HIRD-Net, which integrates both components while still using the SFF stem. This version improved performance to 97.12% (screening) and 76.45% (grading), demonstrating the benefit of combining MSF and MLF. We then redesigned HIRD-Net by replacing the SFF stem with an HFF stem, keeping MSF and MLF intact. This architectural refinement further improved screening accuracy to 97.58% and grading accuracy to 79.74%, highlighting the importance of hierarchical feature extraction early in the network. To evaluate the role of preprocessing and augmentation, we tested baseline models with the proposed enhancement pipeline. Applying image enhancement and augmentation significantly increased grading accuracies to 88.87%, 89.61%, and 90.42% for ResNet-50, DenseNet-201, and InceptionNet, respectively. We evaluated HIRD-Net without image enhancement to isolate the effect of augmentation. It achieved 98.19% screening and 88.16% grading accuracy. The full HIRD-Net model, after incorporating HFF, MSF, MLF, image enhancement, and augmentation, achieved 99.45% screening accuracy and 92.25% grading accuracy. Finally, to explore the impact of the attention mechanism, we introduced the SECA block into the top-performing HIRD-Net version, achieving 99.50% screening accuracy and 93.46% grading accuracy, confirming the synergistic effect of the complete architecture and preprocessing pipeline.

**Figure 15:**
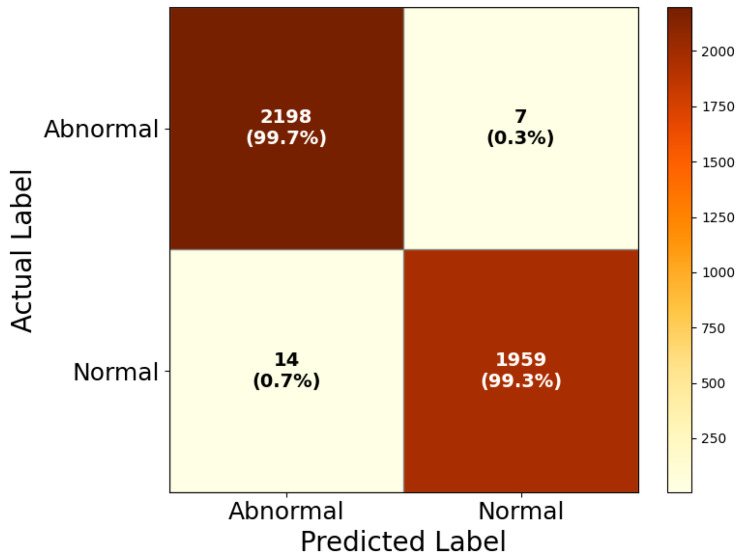
Confusion matrix of HIRD-Net for DR screening.

The performance of the final HIRD-Net for DR screening is illustrated in Figure 15 through its corresponding confusion matrix. The HIRD-Net model successfully classifies 2198 out of 2205 abnormal FIs, yielding a sensitivity of 99.7%, and correctly identifies 1959 out of 1973 normal cases, achieving a specificity of 99.3%. These results indicate extremely low rates of false positives and false negatives, with only 7 abnormal cases incorrectly labeled as normal and 14 normal cases misclassified as abnormal. Such high fidelity in binary classification is critical in medical applications, where missed diagnoses could have severe clinical consequences. The minimal confusion observed between normal and abnormal cases confirms the model’s reliability in distinguishing pathological signs in retinal images.

**Figure 16:**
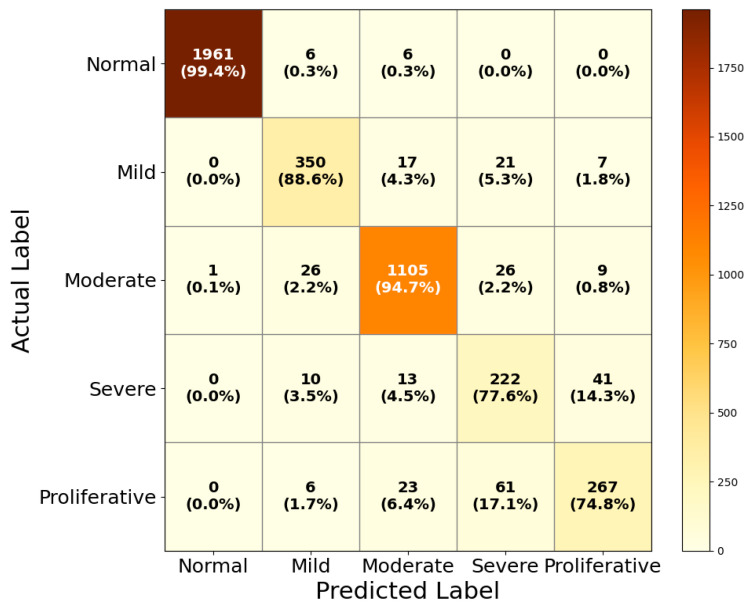
Confusion matrix of HIRD-Net for DR grading.

The results of DR grading, shown in Figure 16, reflect the model’s capacity to handle the more challenging multi-class classification task.

The model achieves 99.4% accuracy in classifying normal images, with only minor confusion seen with mild and moderate DR cases. For mild DR, the classification accuracy reaches 88.6%, though some misclassifications occur with moderate (4.3%) and severe (5.3%) categories, which is expected given the subtle differences in early disease stages. Moderate DR classification yields a high accuracy of 94.7%, again showing minor overlap with adjacent classes. The classification of severe DR stands at 77.6%, with the most frequent confusion arising with proliferative DR (14.3%), reflecting the inherent difficulty in distinguishing between the advanced stages of the disease, where feature overlap is more common. Similarly, the model classifies proliferative DR with an accuracy of 74.8%, with most errors stemming from confusion with severe and moderate DR stages.

**Figure 17:**
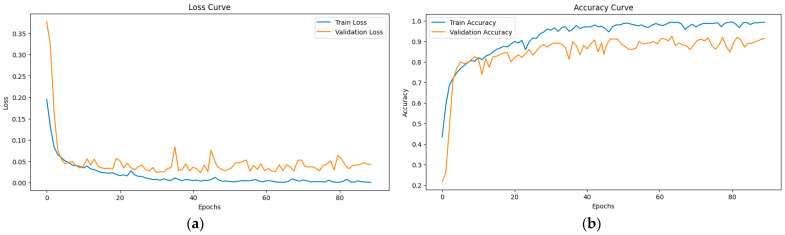
(**a**) Training/validation loss curves; (**b**) training/validation accuracy curves.

To provide a comprehensive evaluation of model convergence and training stability, the training and validation curves for both loss and accuracy in the final DR diagnosis experiment using HIRD-Net are illustrated in Figure 17a,b. These curves reflect consistent optimization behavior and further support the efficacy of the proposed model.

### 4.2. Performance Evaluation on DDR

**Figure 18:**
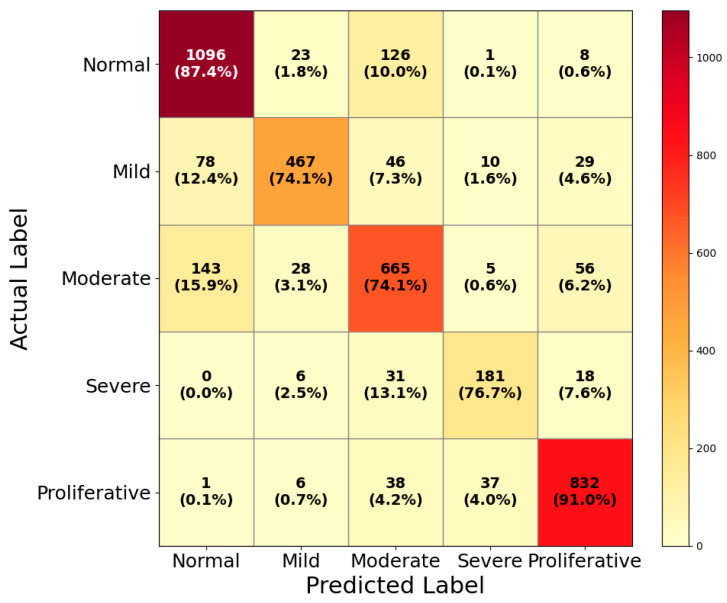
Confusion matrix of HIRD-Net on the DDR dataset.

We utilized the DDR dataset to evaluate HIRD-Net’s generalizability on a diverse collection of retinal images. The multi-class classification results are presented in the confusion matrix in Figure 18.

HIRD-Net correctly classified 1096 normal fundus images, achieving a precision of 83.16%, a recall of 87.40%, and an F1-score of 85.23%. The relatively high recall indicates that the model effectively minimizes false negatives for normal cases, which is essential to avoid unnecessary follow-up for healthy patients. For the Mild DR class, the model achieved a precision of 88.11% and a recall of 74.13%, resulting in an F1-score of 80.52%. While precision is strong, the moderate recall suggests some misclassifications into adjacent stages, likely due to subtle early-stage lesions that resemble normal or moderate DR features. In the Moderate DR category, HIRD-Net achieved a precision of 73.40%, a recall of 74.14%, and an F1-score of 73.77%. This balanced performance reflects the challenge of distinguishing moderate DR from both milder and more severe stages, where overlapping lesion patterns such as microaneurysms and small hemorrhages can lead to confusion. For Severe DR, the model obtained a precision of 77.35%, a recall of 76.69%, and an F1-score of 77.02%. The results indicate consistent performance, though false negatives suggest occasional misclassification into proliferative DR, a common issue in advanced stages due to lesion severity overlap. In the most advanced stage, Proliferative DR, the model achieved the highest performance among the disease classes, with a precision of 88.23%, a recall of 91.03%, and an F1-score of 89.61%. This high recall is clinically valuable, as it ensures that most advanced cases are correctly identified, minimizing the risk of missed diagnoses for patients requiring urgent intervention.

### 4.3. Performance Evaluation on EyePACS

**Figure 19:**
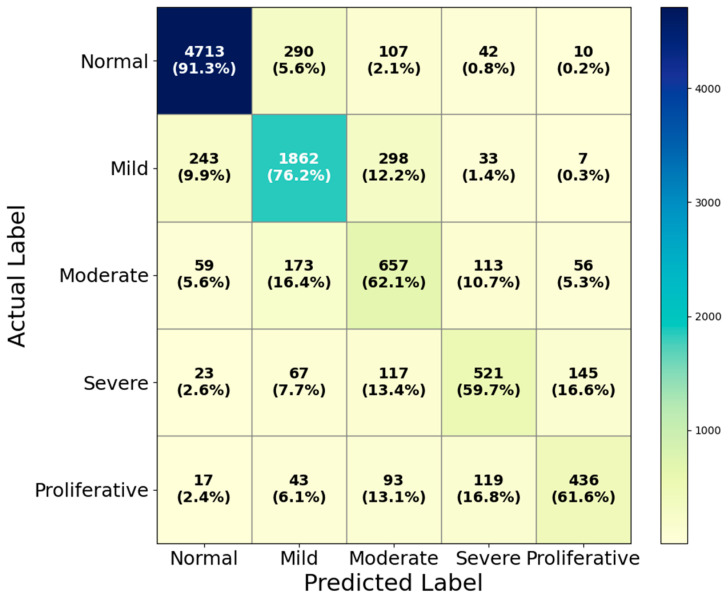
Confusion matrix of HIRD-Net on the EyePACS dataset.

We evaluated HIRD-Net on the EyePACS dataset to further assess its robustness on a large-scale, real-world collection of retinal images exhibiting significant inter-class variability. The multi-class classification results are shown in the confusion matrix in Figure 19. For the Normal class, HIRD-Net achieved the highest performance with a precision of 93.23%, a recall of 91.30%, and an F1-score of 92.26%, indicating strong capability in correctly identifying healthy cases while minimizing false positives. The Mild DR class yielded a balanced precision (76.47%) and recall (76.22%), with an F1-score of 76.34%, suggesting effective detection of early disease signs despite their subtle nature. Performance decreased for Moderate DR, with a precision of 51.65% and recall of 62.10% (F1-score: 56.39%), reflecting the challenge of differentiating mid-stage disease from both adjacent categories due to overlapping lesion features. Severe DR achieved a precision of 62.92% and recall of 59.68% (F1-score: 61.26%), with misclassifications often occurring with proliferative cases. In the most advanced stage, Proliferative DR, the model attained a precision of 66.67%, a recall of 61.58%, and an F1-score of 64.02%, demonstrating reasonable detection performance despite significant intra-class lesion diversity in this dataset.

### 4.4. Grad-CAM-Based Visual Interpretability Analysis of HIRD-Net Predictions

**Figure 20:**
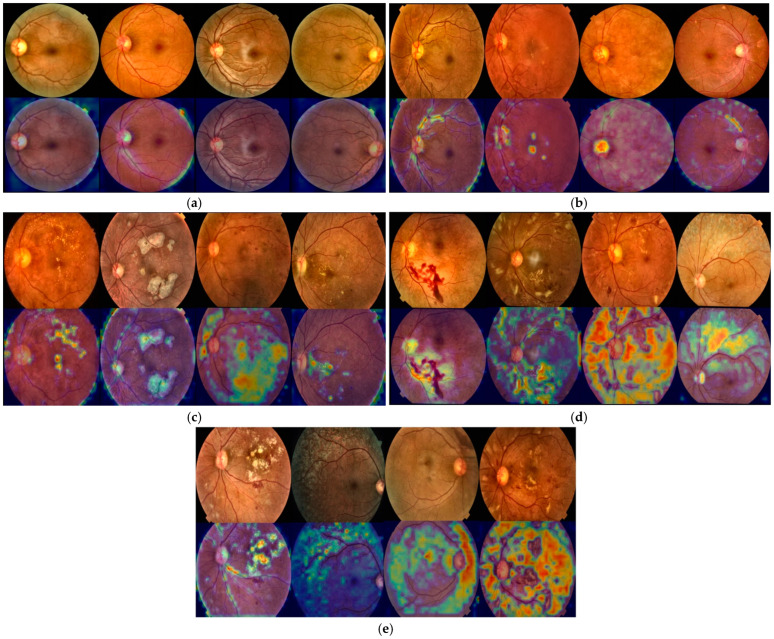
Original images with corresponding Grad-CAM visualizations for: (**a**) Normal, (**b**) Mild, (**c**) Moderate, (**d**) Severe, and (**e**) Proliferative DR classes.

We applied Grad-CAM to HIRD-Net’s final convolutional block, immediately before the fourth global average pooling (GAP) layer, to increase transparency in decision-making and validate the clinical relevance of the learned features. This XAI approach generates class-specific heatmaps that localize the retinal regions most influential to the model’s predictions. By overlaying these heatmaps onto the original FIs, it becomes possible to visually assess whether the model’s attention aligns with known pathological structures such as microaneurysms, hemorrhages, hard and soft exudates, and neovascularization. We analyzed class-wise Grad-CAM visualizations for Normal, Mild, Moderate, Severe, and Proliferative DR cases, enabling qualitative evaluation of model focus and verification of its ability to detect clinically significant features across the full disease spectrum. The Grad-CAM visualizations in Figure 20 provide a class-wise interpretability analysis of HIRD-Net’s predictions across the full DR severity spectrum.

For the Normal class (Figure 20a), no high-intensity activation regions are observed; instead, the model exhibits a diffuse attention pattern focused on the overall retinal structure, vascular integrity, and optic nerve head clarity, confirming the absence of pathological cues and aligning with clinical assessment practices for healthy retinas. In Mild DR cases (Figure 20b), the model highlights variations in the optic nerve head and surrounding vascular structures, with additional localized activations in regions containing microaneurysms or early vascular blockage, indicating its ability to detect subtle pathological features such as minor vessel irregularities and small hemorrhagic spots while retaining broader structural context. For Moderate DR (Figure 20c), Grad-CAM maps reveal strong localized activations around hard exudates and soft exudates (cotton wool spots), with extended focus along affected vascular paths, reflecting the model’s sensitivity to microvascular abnormalities characteristic of disease progression. In Severe DR (Figure 20d), the model’s attention is strongly concentrated on large hemorrhages and dense clusters of hard exudates, showing reduced sensitivity to earlier-stage features, which demonstrates its prioritization of prominent pathological cues during advanced-stage classification. Finally, for Proliferative DR (Figure 20e), HIRD-Net displays extensive, high-intensity activations spanning all four retinal quadrants, with noticeable focus on neovascularization sites, large hemorrhages, and widespread exudative changes; this global attention pattern mirrors clinical diagnostic criteria for advanced disease, highlighting the model’s capacity to capture widespread retinal deterioration.

## 5. Discussion

The comparative analysis and discussion of the experimental results primarily focus on the DR grading performance of various CNN architectures, given that most of the models, including both standard CNNs and the proposed HIRD-Net, achieved near-perfect results on the binary DR screening task. Therefore, we emphasize the comparative analysis of multi-class grading performance, which presents a more challenging and clinically relevant evaluation of a model’s capability to distinguish among the five classes of FIs in DR diagnosis.

### 5.1. Comparative Analysis on IDRiD-APTOS2019 Dataset

We evaluated model performance on the IDRiD-APTOS2019 combined dataset, and the class-wise comparative results in Table 3 reveal consistent performance trends across architectures.

AlexNet demonstrates moderate performance across all DR stages. While it performs adequately for Normal (F1-score = 94.81%) and Moderate (F1-score = 86.18%) classes, it struggles with more advanced stages such as Severe (F1-score = 51.39%) and Proliferative (F1-score = 61.81%). This indicates its limited capacity to capture complex high-level features necessary for differentiating advanced retinal abnormalities.

ResNet18 and ResNet50 show consistent improvements over AlexNet, particularly in Moderate DR grading, achieving F1-scores of 92.85% and 93.02%, respectively. However, they still underperform in Severe and Proliferative stages, where both variants fall below 63% in F1-scores. These results suggest that while residual connections help in stabilizing DL models, their ability to fully capture multiscale retinal pathology remains limited when relying solely on traditional feed-forward architectures. DenseNet201 achieves slightly better performance in the Moderate class (F1-score = 93.58%) and demonstrates noticeable improvement in Mild DR (F1-score = 84.38%) due to its dense connectivity structure that encourages feature reuse. Yet, its performance in Severe (F1-score = 58.76%) and Proliferative (F1-score = 64.15%) stages remains constrained, likely due to insufficient emphasis on multiscale hierarchical patterns within the input images. XceptionNet and InceptionNet V3, both incorporating depth wise separable convolutions and MSF extraction, show marked improvement in classifying Mild, Moderate, and Proliferative DR stages. In particular, InceptionNet V3 achieves an F1-score of 95.27% for Moderate and 81.38% for Mild DR, which affirms the utility of multi-branch convolutional paths in handling spatial variability in lesion manifestations. Nevertheless, both models continue to show weaknesses in Severe DR detection (XceptionNet: F1-score = 57.35%, InceptionNet V3: F1-score = 65.53%), indicating a gap in capturing deeply abstract pathological features. In contrast, the proposed HIRD-Net outperforms all comparative models across nearly every class. It achieves exceptional performance in classifying Normal images with an F1-score of 99.67%, nearly eliminating false positives and negatives. Its performance on Mild (F1-score = 88.27%) and Moderate (F1-score = 94.81%) DR stages confirm that the combined use of hierarchical inception, residual, and dense blocks enables the model to effectively represent features at different levels and scales. Notably, HIRD-Net demonstrates the best F1-score for the Proliferative class (78.41%), significantly outperforming others, and improves on Severe class classification (F1-score = 72.08%), which has historically posed a major challenge for CNNs due to overlapping symptom patterns with adjacent stages. These results highlight the effectiveness of the HIRD-Net framework, which synergizes HFF stem, multi-level and MSF extraction, advanced image enhancement (CLAHE-D-DoG), and class imbalance mitigation through focal loss. As a result, HIRD-Net achieves superior DR grading performance with enhanced sensitivity and precision, particularly in clinically critical stages such as Severe and Proliferative DR.

**Figure 21:**
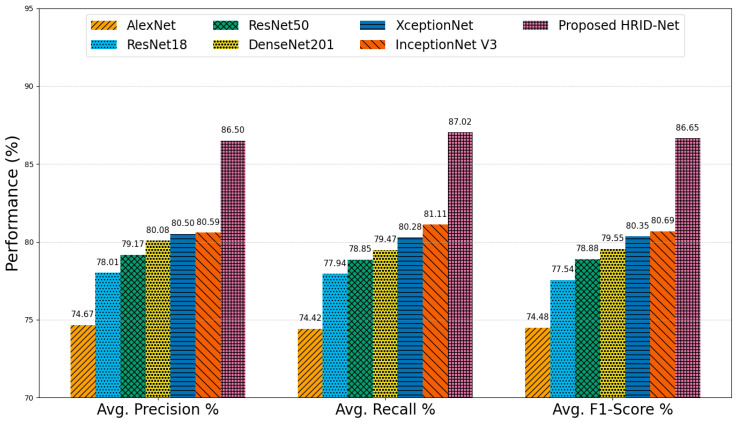
Average performance comparison on the IDRiD-APTOS2019 dataset.

The average performance metrics for DR grading across various CNN models are comprehensively illustrated in Figure 21.

These metrics include average precision, recall, and F1-score, which collectively reflect the models’ ability to accurately detect and classify different stages of DR. Starting with AlexNet, the baseline performance across all three metrics remains the lowest, with 74.67% precision, 74.42% recall, and a corresponding F1-score of 74.48%. This suggests that while AlexNet can perform basic classification, its shallow architecture and lack of specialized feature extraction modules limit its effectiveness in handling the complex variability found in DR lesions. ResNet18 and ResNet50, which incorporate residual connections to address vanishing gradients and enable deeper learning, show progressive improvement. ResNet18 achieves an average precision of 78.01%, a recall of 77.94%, and an F1-score of 77.54%. ResNet50 improves upon these results with 79.17% precision, 78.85% recall, and 78.88% F1-score, indicating a better balance between sensitivity and specificity due to its deeper architecture and MLF extraction. DenseNet201, known for its dense connectivity and efficient feature reuse, further enhances the results, achieving 80.08% precision, 79.47% recall, and an F1-score of 79.55%. This improvement underscores the benefits of densely connected layers in capturing intricate patterns from FIs. The performance of XceptionNet and InceptionNet V3 is slightly superior, leveraging MSF extraction through depth-wise separable convolutions and inception modules, respectively. XceptionNet reaches 80.50% precision, 80.28% recall, and 80.35% F1-score, while InceptionNet V3 records 80.59%, 81.11%, and 80.69% for the same metrics. Their robust spatial features handling capabilities make them more suitable for fine-grained retinal analysis. Among all models, the proposed HIRD-Net clearly outperforms its counterparts, with an average precision of 86.50%, a recall of 87.02%, and an F1-score of 86.65%. This superior performance is attributed to its carefully designed architecture, which integrates MLF and MSF, a hierarchical feature fusion (HFF) stem, and a robust preprocessing pipeline that includes contrast enhancement and data augmentation. These components work synergistically to improve the model’s ability to capture both local and global retinal features, enabling accurate classification of DR severity levels.

### 5.2. Comparative Analysis on DDR Dataset

**Figure 22:**
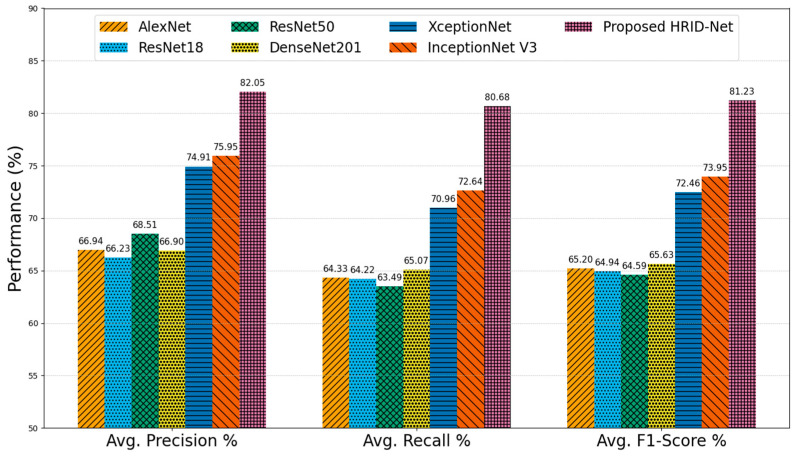
Average performance comparison on the DDR dataset.

We used the DDR dataset, a large, diverse collection of retinal images, to assess each architecture’s generalizability to heterogeneous clinical data. Figure 22 shows clear trends across models.

AlexNet, ResNet18, and ResNet50 achieved average F1-scores of 65.20%, 64.94%, and 64.59%, respectively, indicating that deeper residual connections in ResNet50 offer improved feature extraction over AlexNet; the absence of advanced multi-scale modeling limits performance in complex, real-world lesion presentations. DenseNet201 produced a comparable F1-score of 65.63%, benefiting from dense connectivity for feature reuse but still lacking sufficient hierarchical representation to address lesion scale variability. XceptionNet and InceptionNet V3 demonstrated notable improvements, with F1-scores of 72.46% and 73.95%, respectively, due to their depthwise separable convolutions and multi-branch processing, which enhance the capture of spatially diverse lesion patterns. However, these models still struggled to maintain high recall in advanced DR stages. In contrast, the proposed Attention HIRD-Net achieved the highest overall performance with an average precision of 82.05%, recall of 80.68%, and F1-score of 81.23%.

### 5.3. Comparative Analysis on EyePACS Dataset

**Figure 23:**
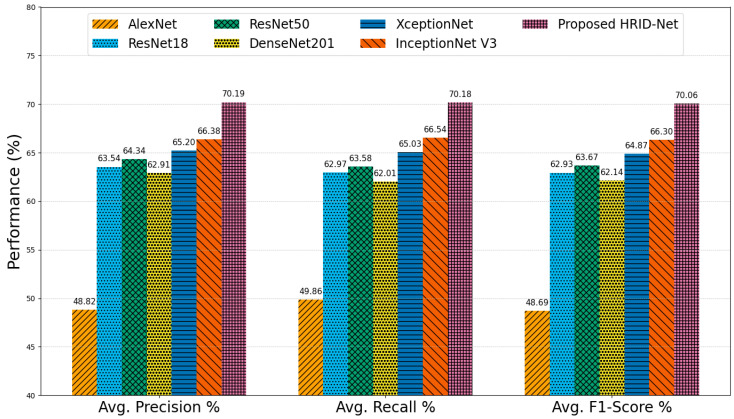
Average performance comparison on the EyePACS dataset.

The EyePACS dataset, characterized by its large scale, high variability in image quality, and high class imbalance, presents a challenging benchmark for DR grading. Figure 23 summarizes the average performance of state-of-the-art models on the EyePACS dataset. AlexNet recorded the lowest performance, with 48.82% precision, 49.86% recall, and a corresponding F1-score of 48.69%, reflecting its inability to generalize to the dataset’s diversity due to its shallow architecture and limited feature extraction capacity. ResNet18 (precision = 63.54%, recall = 62.97%, F1 = 62.93%) and ResNet50 (precision = 64.34%, recall = 63.58%, F1 = 63.67%) demonstrated clear improvements, benefiting from deeper residual connections that enhance representational power. However, both models showed nearly balanced precision and recall values, suggesting they are equally prone to false positives and false negatives in challenging cases. DenseNet201 achieved similar averages (precision = 62.91%, recall = 62.01%, F1 = 62.14%), indicating that while dense connectivity improves feature reuse, it does not substantially address variability in lesion scale or poor contrast conditions. XceptionNet improved precision to 65.20% and recall to 65.03% (F1 = 64.87%), while InceptionNet V3 further increased these metrics to 66.38%, 66.54%, and 66.30%, respectively demonstrating the benefits of multi-branch architectures for capturing MSF, though still limited by the dataset’s inherent noise and imbalance. The proposed Attention HIRD-Net achieved the highest overall performance, with a precision of 70.19%, a recall of 70.18%, and an F1-score of 70.06%, representing a balanced and substantial improvement across all metrics. This consistency indicates that the model not only detects DR lesions more accurately but also maintains robust sensitivity by leveraging a combination of hierarchical feature fusion, multi-scale processing, attention-based refinement, and advanced preprocessing strategies.

### 5.4. Overall Comparative Evaluation

The overall classification accuracy and model complexity, measured in terms of the number of trainable parameters (in millions), are critical indicators of the effectiveness of DL models. Table 4 comparison among popular CNN architectures and the proposed HIRD-Net reveals insightful observations regarding the performance and computational cost.

**Table 4:** Overall accuracy and model complexity comparison of CNN architectures.

Framework	CNN Architecture	IDRiD-APTOS2019 Accuracy (%)	DDR Accuracy (%)	EyePACS Accuracy (%)	Parameters (Million)
[31]	AlexNet	84.97	71.13	58.77	~61
[47]	ResNet18	88.75	71.92	73.39	~11.7
[25,37,38]	ResNet50	88.87	72.73	74.39	~25.6
[34]	DenseNet201	89.61	74.46	74.09	~20.2
[48]	XceptionNet	90.16	76.88	74.92	~22.9
[14,31]	InceptionNet V3	90.43	78.28	76.02	~23.8
Proposed	HIRD-Net	93.46	82.45	79.94	~4.8

AlexNet achieves an overall accuracy of 84.97%, 71.13% and 58.77% of all three datasets with approximately ~61 million parameters, making it the most parameter-heavy yet the least accurate among the models compared. This highlights the inefficiency of simple feed-forward architectures, which, despite their large size, lack the advanced feature extraction capabilities necessary for effective DR diagnosis. A MLF-based ResNet18 model with ~11.7 million parameters, delivers a significantly better accuracy of 88.75%, 71.92% and 73.39% demonstrating the advantage of residual learning. Its deeper variant, ResNet50, with ~25.6 million parameters, marginally improves the accuracy to 88.87%, 72.73%, and 74.39% suggesting diminishing returns in performance with increased depth unless accompanied by specialized architectural improvements. DenseNet201, with ~20.2 million parameters, achieves a higher accuracy of 89.61%, 74.46%, and 74.09% leveraging dense connectivity for better gradient flow and feature reuse. Similarly, multiscale models XceptionNet and InceptionNet V3 achieve accuracies of 90.16%, 76.88%, 74.92% and 90.43%, 78.28%, 76.02% with ~22.9 and ~23.8 million parameters, respectively. These models utilize depthwise separable convolutions and inception modules to capture multi-scale spatial features, offering a better accuracy-to-parameter ratio compared to traditional architectures. Remarkably, the proposed HIRD-Net attains the highest accuracy of 93.46%, 82.45%, and 79.94% on all three datasets while maintaining the lowest parameter count of just ~4.8 million. This reflects a major architectural advancement, demonstrating that with intelligent design incorporating HFF stem, MSF, MLF blocks, and optimized preprocessing, results in significant performance gains with minimal computational overhead.

## 6. Conclusions and Future Work

This research introduced an efficient deep learning framework, HIRD-Net, for DR diagnosis using enhanced FIs. The integration of CLAHE and D-DoG filtering in the preprocessing pipeline significantly improved lesion visibility and local contrast. The hierarchical feature fusion architecture enabled the retention of critical information across multiple network depths, ensuring that fine details such as microaneurysms, which often vanish in deeper layers, were preserved from the Normal to Mild DR stages, thereby minimizing information loss in early disease detection. The multi-scale modules proved particularly effective for advanced DR stages (Severe and Proliferative), capturing broad regions of interest and accommodating the spatial diversity of large hemorrhages and neovascularization. The inclusion of SECA blocks further refined the learned representations by adaptively emphasizing lesion-relevant channels while suppressing irrelevant features, enhancing discriminative capacity across all DR classes. The use of the Hard-Swish activation function improved gradient flow stability and effectively handled dataset nonlinearities, while the focal loss function mitigated class imbalance, enabling robust classification across severity levels. The proposed pipeline achieved high accuracy with low computational overhead, making it well-suited for deployment in clinical environments. Finally, we employed Grad-CAM as an XAI tool to visualize the model’s decision-making process. The heatmaps generated confirmed that HIRD-Net’s attention consistently aligned with clinically significant retinal lesions, thereby enhancing interpretability and supporting trust in automated DR diagnosis. Overall, the synergy between targeted preprocessing, multi-level and multi-scale representation learning, attention-based refinement, and interpretability tools positions HIRD-Net as a reliable, efficient, and clinically relevant solution for DR screening and grading.

Although HIRD-Net shows strong potential, some limitations remain, particularly in differentiating between adjacent DR severity levels due to overlapping visual characteristics and the lack of localized lesion annotations. To address this, future work may incorporate quadrant-based image partitioning and region-specific labeling to facilitate more precise learning. Incorporating multimodal data such as OCT [19], patient demographics, or clinical history may further improve classification robustness. Additionally, the preprocessing and feature extraction techniques demonstrated in this work are transferable to other medical imaging domains, including dermatology, radiography, and histopathology, where contrast enhancement and spatial context are essential. Exploring hybrid models that combine CNNs with stacked auto-encoders [49] or attention-based transformer [5] architectures could also enhance performance, particularly in complex or heterogeneous datasets. These directions offer promising pathways to extend the applicability and impact of the proposed framework.

## Figures and Tables

**Table 1:** Dataset composition after preprocessing and augmentation.

	Before Augmentation			After Augmentation		
Classes	IDRiD + APTOS2019	DDR	EyePACS	IDRiD + APTOS2019	DDR	EyePACS
Normal	1973	6266	25,810	9865	6266	25,810
Mild DR	395	630	2443	1975	3150	12,215
Moderate DR	1167	4477	5292	5835	4477	5292
Severe DR	286	236	873	1430	1180	4365
Proliferative DR	357	913	708	1785	4565	3540

**Table 2:** Ablation study showing the effect of architectural components.

Model	Enhancement	Augmentation	Stem	MLF	MSF	Attention Block	Screening Accuracy %	Grading Accuracy %
ResNet50	✘	✘	SFF	✔	✘	✘	93.13	61.79
DenseNet	✘	✘	SFF	✔	✘	✘	95.88	68.56
InceptionNet	✘	✘	SFF	✘	✔	✘	96.72	72.01
HIRD-Net	✘	✘	SFF	✔	✔	✘	97.12	76.45
HIRD-Net	✘	✘	HFF	✔	✔	✘	97.58	79.74
ResNet50	✔	✔	SFF	✔	✘	✘	97.27	88.87
DenseNet	✔	✔	SFF	✔	✘	✘	98.03	89.61
InceptionNet	✔	✔	SFF	✘	✔	✘	97.93	90.42
HIRD-Net	✘	✔	HFF	✔	✔	✘	98.19	88.16
HIRD-Net	✔	✔	HFF	✔	✔	✘	99.45	92.25
HIRD-Net	✔	✔	HFF	✔	✔	✔	99.50	93.46

**✘**: Indicates that the component is excluded; **✔**: Indicates that the component is incorporated; SFF: Standard feed-forward feature extraction stem; HFF: Hierarchical feature fusion-based stem; MLF: Integration of multi-level feature representations; MSF: Utilization of multi-scale feature extraction strategies.

**Table 3:** Class-wise performance comparison of CNN models for DR diagnosis.

CNN Architecture	Class	TP	FP	FN	TN	Pr. (%)	Rec. (%)	F1. (%)
Alex-Net	Normal	1871	103	102	2307	94.8	94.8	94.81
	Mild	303	77	92	3875	79.7	76.7	78.19
	Moderate	1007	163	160	3171	86.1	86.3	86.18
	Severe	157	168	129	4021	48.3	54.9	51.39
	Proliferative	212	117	145	3966	64.4	59.4	61.81
ResNet18	Normal	1965	81	8	2213	96.0	99.6	97.79
	Mild	326	80	69	3852	80.3	82.5	81.40
	Moderate	1045	39	122	3133	96.4	89.5	92.85
	Severe	199	170	87	3979	53.9	69.6	60.76
	Proliferative	173	100	184	4005	63.4	48.5	54.92
ResNet50	Normal	1927	82	46	2251	95.9	97.7	96.79
	Mild	315	62	80	3863	83.6	79.7	81.61
	Moderate	1073	67	94	3105	94.1	91.9	93.02
	Severe	193	141	93	3985	57.8	67.5	62.26
	Proliferative	205	113	152	3973	64.5	57.4	60.74
DenseNet201	Normal	1937	88	36	2241	95.7	98.2	96.90
	Mild	362	101	33	3816	78.2	91.6	84.38
	Moderate	1072	52	95	3106	95.4	91.9	93.58
	Severe	161	101	125	4017	61.5	56.3	58.76
	Proliferative	212	92	145	3966	69.7	59.4	64.15
XceptionNet	Normal	1928	42	45	2250	97.9	97.7	97.79
	Mild	356	47	39	3822	88.3	90.1	89.22
	Moderate	1094	82	73	3084	93.0	93.7	93.38
	Severe	156	102	130	4022	60.5	54.5	57.35
	Proliferative	233	138	124	3945	62.8	65.3	64.01
InceptionNet V3	Normal	1925	41	48	2253	97.9	97.6	97.74
	Mild	343	105	52	3835	76.6	86.8	81.38
	Moderate	1107	50	60	3071	95.7	94.9	95.27
	Severe	193	110	93	3985	63.7	67.5	65.53
	Proliferative	210	94	147	3968	69.1	58.8	63.54
Proposed HIRD-Net	Normal	1961	1	12	2217	99.9	99.4	99.67
	Mild	350	48	45	3828	87.9	88.6	88.27
	Moderate	1105	59	62	3073	94.9	94.7	94.81
	Severe	222	108	64	3956	67.3	77.6	72.08
	Proliferative	267	57	90	3911	82.4	74.8	78.41

## Data Availability

The datasets used in this research are publicly available and have been cited within the manuscript.
